# Between wellness and fairness: The mediating role of autonomous human choice and social capital in OECD countries

**DOI:** 10.1002/jcop.22822

**Published:** 2022-02-16

**Authors:** Salvatore Di Martino, Michael P. Scarpa, Isaac Prilleltensky

**Affiliations:** ^1^ Department of Psychology University of Bradford Bradford UK; ^2^ Department of Educational and Psychological Studies University of Miami Coral Gables Florida USA

**Keywords:** autonomous human choice, GDP, happiness, life satisfaction, Manifest Path Analysis, social capital, social justice

## Abstract

Theoretical arguments and empirical evidence have been provided in the literature for the role of fairness in wellness. In this paper, we explore the role of two potential mediating variables: autonomous human choice and social capital. Using aggregated panel data across countries belonging to the Organisation for Economic Co‐operation and Development (OECD), we compared the OECD Social Justice Index (SJI) with data on life satisfaction to test whether fairness has direct and indirect effects on wellness. Results from a series of Manifest Path Analyses with time as fixed effect, support the hypothesis that the OECD SJI is directly linked to country‐level life satisfaction, additionally revealing that its indirect effect operates primarily through people's autonomous choices in life and their country's level of social capital. Our results contribute to two distinct bodies of knowledge. With respect to community psychology, the findings offer empirical evidence for the synergistic effect of personal, relational, and collective factors in well‐being. With respect to the impact of economic inequality on wellness, we extend the literature by using social justice as a more comprehensive measure. Limitations and recommendations for future studies are discussed.

## 1 INTRODUCTION

Wellness, a key construct in community psychology (CP), has been defined as “a positive state of affairs, brought about by the simultaneous and balanced satisfaction of diverse objective and subjective needs of individuals, relationships, organizations, and communities” (Prilleltensky, 2012, p. 2). Fairness, another foundational tenet of CP, has been linked to wellness at multiple levels. Recent empirical work demonstrates that this is the case at the interpersonal (Hirigoyen, 2000; Kelly et al., 2009; Sutton & Douglas, 2005), occupational (De Vogli et al., 2007; Elovainio et al., 2002), communal (Adler, 2009; Adler & Stewart, 2010), and societal levels (Di Martino & Prilleltensky, 2020). It has also been suggested in CP that wellness or well‐being are the results of the synergy of three important factors: personal, relational, and communal. Personal factors include empowerment, sense of control, competency, and resilience. Relational factors include empathy, compassion, inclusion, social support, social capital, and psychosocial accompaniment. Communal factors, in turn, entail social justice, fairness, equity, and equality (Prilleltensky, 1997, 2001; Riemer et al., 2020; Watkins, 2019). In CP, that synergy has been well established by researchers who have explored the role of social capital and autonomy in well‐being (Perkins et al., 2002; van Uchelen, 2000), and others who have suggested alternatives to the gross domestic product (GDP) as a measure of progress in societies (Natale et al., 2016). These efforts notwithstanding, the interconnected nature of these variables requires considerable integration. Although some studies offer empirical evidence supporting the synergistic effect of these factors at the personal and organizational levels, they have mostly addressed them from an individual or at the very best community perspective; a national perspective is still missing.

This means that we do not yet have as much evidence of the combined effect of personal (autonomy), relational (social capital), and collective (social justice) factors on wellness. What we aim to show is that the complex relationship between them can be better understood if we analyze the primary role of social justice (Prilleltensky, 2001).

Social justice policies and practices aim to provide each individual *his or her due* (Sandel, 2020), where *due* refers not only to material goods such as money or housing, but also subjective goods such as respect, voice, and choice. In psychology, it is common to assess subjective perceptions of justice and fairness, but in this paper, we are interested in social policies that advance the cause of justice, not in people's observations about the fairness of social arrangements. In this study, we expand on what is already known about the impact of social justice on wellness. We do so by considering the additional roles of autonomy and relatedness. There are two foundational theories that guide our effort. The first is the capabilities approach articulated by Nussbaum and Sen (1993). This framework understands well‐being as a function of the ability of individuals to do what they desire and become who they want to be. This model, widely embraced in CP (Munger et al., 2016; Shinn, 2015), emphasizes the autonomy of individuals to realize their potential, and their dependence on others to do so (relatedness).

The second theory guiding our effort is the psychology of mattering. Recent theoretical and empirical work has shown that mattering consists of feeling valued and adding value (Prilleltensky & Prilleltensky, 2021; Scarpa, Di Martino, et al., 2021; Scarpa, Prilleltensky, et al., 2021). Part of adding value is autonomy, and part of feeling valued is relatedness. Based on that theory, we surmise that in countries where there are higher degrees of autonomy and relatedness, people will report higher levels of well‐being, since mattering is highly correlated to well‐being (Flett, 2018; Prilleltensky & Prilleltensky, 2021; Scarpa, Zopluoglu, et al., 2021).

To summarize, our argument derives from three lines of thought. First, the theory of mattering (Prilleltensky & Prilleltensky, 2021) and the capabilities approach (Nussbaum, 2011; Nussbaum & Sen, 1993) emphasize the roles of autonomy and relatedness in promoting well‐being. Second, considerable work has shown that fairness is a precondition for wellness (Prilleltensky, 2012). Third, there is growing evidence at the personal level that mattering mediates between wellness and justice (Scarpa, Di Martino, et al., 2021). When we combine these bodies of knowledge, we conclude that autonomy and relatedness may very well mediate between fairness and wellness at the national level.

The Organisation for Economic Co‐operation and Development (OECD) presents an interesting opportunity to study the role of fairness, autonomy, and relatedness on wellness since, as a group of nations, it is invested in studying and promoting well‐being policies (OECD, 2020). Recent findings show that income inequality, for instance, has consistently deteriorated from 2010 to 2018 (OECD, 2020). Interestingly, their recent work does not include a comprehensive measure of social justice, which is one of our contributions in the present study.

In summary, the present study seeks to enrich our understanding of the roles of fairness, autonomy, and relatedness on wellness. In doing so, we aim to contribute to at least two distinct bodies of knowledge: the synergy of personal, relational, and collective factors in CP, and the epidemiology of well‐being across nations. Our review of the literature led us to devise a model that incorporates fairness, autonomy, and relatedness via several directional hypotheses. These will be outlined in the next section.

### 1.1 The relationship between social justice, social capital, and life satisfaction

Our investigation stems from the notion that wellness is produced by experiences of fairness and conditions of justice across multiple domains of life (Di Martino & Prilleltensky, 2020; Nussbaum, 2002; Prilleltensky, 2012; Wilkinson & Pickett, 2018). Despite this well‐established relationship, the specific pathways producing this connection are unclear (Venkatapuram, 2021), especially at the national level. Social capital represents one such potential pathway. The definition and meaning of social capital are somewhat contested (Fulkerson & Thompson, 2008; Putnam & Romney Garrett, 2020), yet the literature acknowledges that it entails social networks, civic engagement, norms of reciprocity, and generalized trust (Bhandari & Yasunobu, 2009). As such, it meets our definition of relatedness.

The literature supports a link between at least some elements of social capital—particularly social trust—and several aspects of wellness (Putnam & Romney Garrett, 2020) and social justice (Cozzolino, 2011). These include equality/inequality (Ferragina, 2010), quality of governance and government performance (Boix & Posner, 1998), and democracy (Newton, 2001; Paxton, 2002). Theorists have suggested that social capital may be a mediator between inequality and health disparities (Kawachi & Kennedy, 1997), mirroring the proposed role it plays in our model as a mediator between social justice and life satisfaction. Both psychosocial (e.g., trust, social comparison) and public health (e.g., investment in health services) explanations have been put forward for this association (Hawe & Shiell, 2000). Experimental and correlational studies have provided evidence that conditions of injustice, inequality, and unfairness lower multiple aspects of social capital, including generalized trust (Cleaver, 2005).

Meanwhile, numerous studies have investigated the relationship between social capital and life satisfaction, generally finding a significant link between the two constructs. Pugno and Verme (2012) reviewed several studies, concluding that social capital “contributes greatly… to explaining the cross‐country variance of individual life satisfaction” (p. 4). Specifically, they note that time spent involved in social engagement predicts life satisfaction, whereas declining social capital is associated with decreasing happiness.

While the links between inequality and social capital, and between social capital and well‐being respectively, appear well supported, research into the full mediation pathway has featured inconsistent findings (Layte, 2012; Vilhjalmsdottir et al., 2016). This suggests a need for further research.

### 1.2 The relationship between social justice, autonomous human choice, and life satisfaction

Cultural psychology has long acknowledged that societies are driven by different sets of values. Some, for example, tend to encourage individual efforts whereas others promote collective achievements. This distinction has been conceptualized in terms of individualism versus collectivism (Hofstede et al., 2010), autonomy versus embeddedness (Schwartz, 2009), and self‐expression versus survival (Inglehart, 1997). Despite some theoretical differences, individualism, autonomy, and self‐expression are very much related in that they all express a societal inclination to promote individual choice, personal freedom, and self‐actualization (Inglehart & Oyserman, 2004). In fact, according to Inglehart and Welzel (2005), these three constructs “reflect a common theme: an emphasis on autonomous human choice” (p. 136).

Given this similarity, we adopted autonomous human choice as a potential mediator between social justice and life satisfaction. As described above, the capabilities approach and the theory of mattering provide the basic theoretical rationale for this mediation relationship. Nussbaum (2002) illustrates this pathway through a discussion of women's caretaking responsibilities. Because of “unequal social and political circumstances” (p. 124), women “need to spend long hours caring for the physical needs of others” which “makes it difficult to do what they want to do in other areas of life” (p. 125). Importantly, “what they want to do” includes meeting basic physical, psychological, and social needs. This example illustrates the basic theory underlying our hypothesis: unjust social situations create extra demands and obstacles for some members of society. These obstacles and demands inhibit autonomy required to meet basic needs and, in so doing, inhibit life satisfaction. Similarly, according to mattering theory, unjust social arrangements interfere with the ability of people to add value to themselves and society (Prilleltensky & Prilleltensky, 2021).

A similar theoretical pathway is articulated by Anderson and Honneth (2005), who describe autonomy as an intersubjective, socially supported sense of the capacity to lead one's own life. The role of social justice in liberal societies in their account implies a commitment to reducing “autonomy‐related vulnerabilities” (p. 127). Under such a scheme, greater social justice would be expected to lead directly to increased autonomy by better fostering the conditions that support it.

Such an understanding would also align with the self‐determination theory (SDT), according to which autonomy supports well‐being (Ryan & Deci, 2017; Deci & Ryan, 2013). Empirically, there is ample support for this perspective. One study grounded in SDT found that supporting autonomy strongly predicted life satisfaction in Mexican, Brazilian, and Argentinian samples (Cavazos Arroyo, 2013). This finding corroborates earlier results suggesting that autonomy predicts well‐being across cultures (Wichmann, 2011). Others have found a clear contribution to well‐being from perception of primary goods including freedom of movement and occupation, basic liberties, and sufficient financial resources to enable various ends (Bradshaw et al., 2021).

### 1.3 The relationship between social justice, GDP, and life satisfaction

GDP represents another potential mediator between social justice and life satisfaction. The empirical relationship between GDP and life satisfaction is well established (Diener & Diener, 1995; Hellmann et al., 2019; United Nations, 2006). Researchers have also suggested that social justice improves economic productivity by creating conditions for greater employment access and enhanced information sharing (Kitson et al., 2000; Stiglitz, 2002). Some empirical evidence supports a directional connection. For instance, in a working paper, Kavuri and Shao (2017) demonstrate a strong correlation between national‐level social justice as measured by an expert‐rated index and GDP among developed nations, even when controlling for endogeneity as well as cultural, linguistic, and geographic factors.

In summary, we find sufficient evidence in the literature to support the hypotheses that autonomous human choice and relatedness mediate between conditions of fairness and outcomes of wellness.

## 2 METHOD

Based on the preceding rationale, in this study, we tested two hypotheses.

Hypothesis 1 In continuity with previous findings (Di Martino & Prilleltensky, 2020), our first hypothesis was to test through an OLS multiple regression analysis whether the OECD Social Justice Index (SJI) predicted national life satisfaction, after controlling for well‐established macro‐level covariates (i.e., social capital, GDP, autonomous human choice, postcommunism), and the effect of time.

Hypothesis 2 The second hypothesis assumes that autonomy, social capital, and GDP are mediators of the relationship between the OECD SJI and life satisfaction. This hypothesis rests on both theoretical and statistical grounds, detailed above and briefly reviewed here.

This second hypothesis was tested through a Path Analysis with manifest variables. Given the presence of univariate non‐normality in some of our variables (see histograms in Appendix A) and multivariate non‐normality (Mardia skewness *χ* ^2^ = 491.78, *p* < 0.001, Mardia kurtosis, *χ* ^2^ = 15.10, *p* < 0.001), maximum likelihood with 1000 Bollen–Stine corrected bootstraps (Bollen & Stine, 1992) was implemented to estimate *χ*2 test of model fit parameters. For the other indices of model fit available in Lavaan, we considered the following conventional standards (Hu & Bentler, 1999): comparative fit index (CFI) and Tucker–Lewis index (TLI) > 0.95, root mean square error of approximation (RMSEA) < 0.05, and standardized root mean square residual (SRMR) < 0.08. In accordance with literature guidelines (Bollen & Stine, 1990), we estimated standardized standard errors, *p*‐value, and 95% confidence intervals from 1000 Bollen–Stine corrected bootstraps to test for indirect and total of effect of mediated pathways.

### 2.1 Social justice

**Figure 1:**
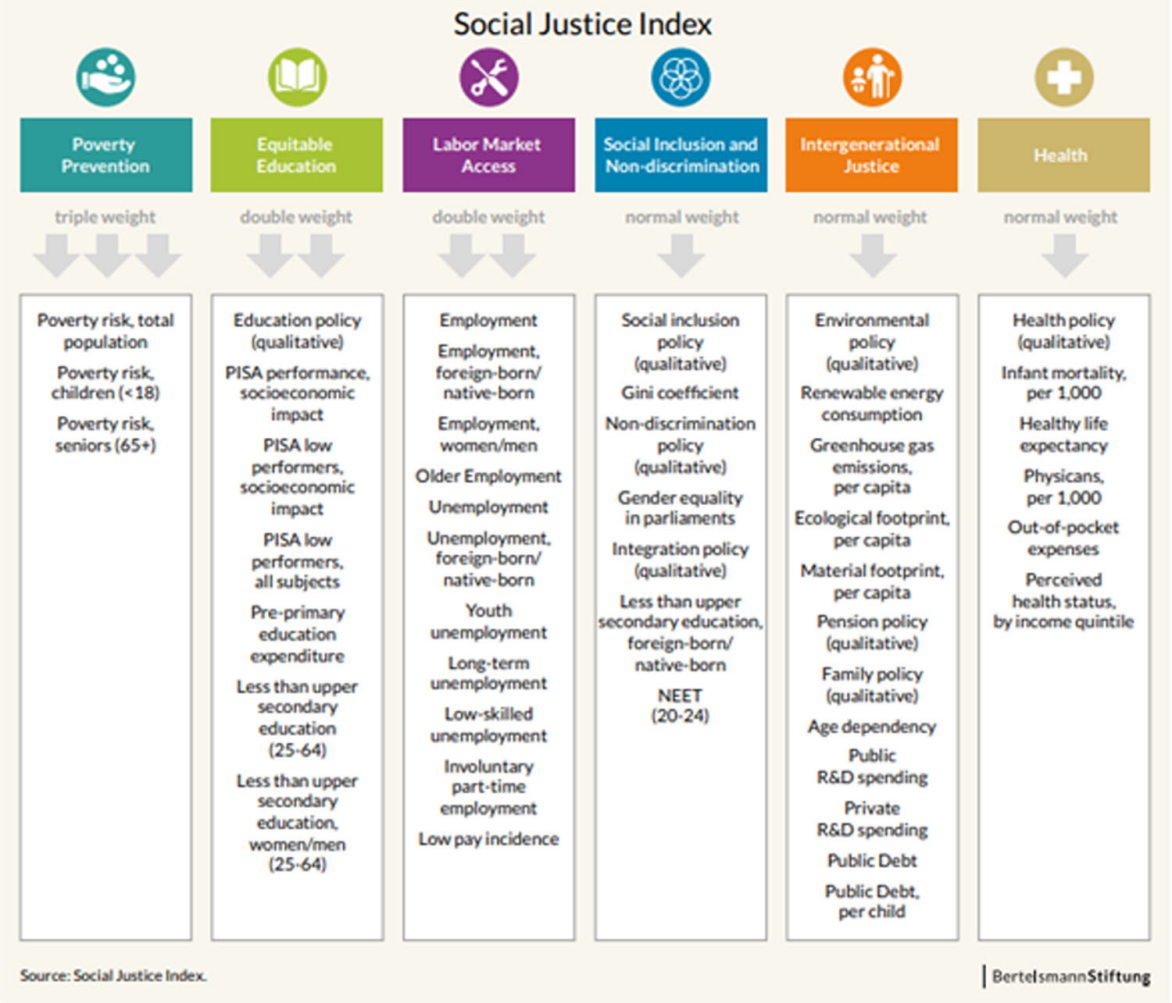
Domains and indicators of the Organisation for Economic Co‐operation and Development (OECD) Social Justice Index. Source: Reproduced from Hellmann et al. (2019)

In this study, social justice was operationalized through the OECD SJI, which was developed by the Bertelsmann Stiftung Institute. The OECD‐SJI aims “to deliver a conceptually cohesive and empirically meaningful overall ranking of all OECD and EU member states and measures on a regular basis the progress made, and the ground lost on issues of social justice” (Hellmann et al., 2019, p. 130). The index shares several features with the EU SJI (Schraad‐Tischler et al., 2017). Both indexes rest on the same theoretical ground, drawing from Rawls' (1999) theory of justice—particularly the concept of equal opportunities—and the capabilities approach (Nussbaum & Sen, 1993). They also follow the approach and procedure derived from Merkel (2001, 2007) and Merkel and Giebler (2009) to measure the following six domains of social justice: poverty prevention, equitable education, labor market access, social inclusion and nondiscrimination, intergenerational justice, and health (see Figure 1). For more information about the theoretical and methodological aspects of the OECD SJI, we refer the readers to the index report (Hellmann et al., 2019).

Compared to the EU SJI, its OECD counterpart includes a total of 38 quantitative indicators and 8 qualitative indicators. While the latter has remained the same, some quantitative indicators such as “early school leavers,” “employment disabled/nondisabled,” “discrimination against people with disabilities,” and “health system accessibility and range” have been dropped in the OECD version “either because of insufficient available data or because there were problems of data comparability between different sources” (p. 135). On the other hand, the OECD SJI includes new indicators such as “poverty risk, children (<18),” “ecological footprint per capita,” and “infant mortality per 1000.” Lastly, the OECD SJI covers eight time points (i.e., 2009, 2011, and from 2014 until 2019), compared to the six of the EU version.

### 2.2 Life satisfaction

Following the results of an earlier study (Di Martino & Prilleltensky, 2020), we treated life satisfaction as our main dependent variable. In this study, we use life satisfaction as one of the main elements of individual wellness.

Data on self‐reported life satisfaction were sourced from the *World Happiness Report 2019* (Helliwell et al., 2019), which provides country‐level aggregated data from the Gallup World Poll (GWP). The GWP has been tracking global and relevant issues, including life satisfaction, since 2005 by interviewing people in more than 160 countries worldwide and in over 140 languages (http://www.gallup.com/178667/gallup-world-poll-work.aspx). Life satisfaction is based on the answers to the following question: “Please imagine a ladder, with steps numbered from 0 at the bottom to 10 at the top. The top of the ladder represents the best possible life for you and the bottom of the ladder represents the worst possible life for you. On which step of the ladder would you say you personally feel you stand at this time?”

### 2.3 Mediators and controlling variables

This study examines a previously tested set of variables (Di Martino & Prilleltensky, 2020), which have proven to be relevant predictors of national life satisfaction (see Bjørnskov, 2003; Bjørnskov et al., 2008; Kim & Kim, 2012). Our additional aim was to cover a wide range of societal aspects connected to national happiness, including economic (GDP), relational (social capital), cultural (autonomy), and political (postcommunism) factors. We briefly describe the key variables in the study. As we will better see in the next sections, we also treated some variables as mediators of the relationship between social justice and wellness.

In terms of GDP, we used GDP per capita, PPP (current international $), which assigns each country analyzed equal purchasing power in international dollars.

Social capital was measured by the Legatum Institute (https://li.com/), which has developed a comprehensive index of social capital which “measures how cohesive a society is in terms of people trusting, respecting and helping one another, and the institutional structures they interact with” (Legatum Institute, 2019, p. 48). The index includes the following sub‐domains: personal and family relationships, social networks, interpersonal trust, institutional trust, and civic and social participation.

For this study, we chose to collect data that reflect the concept of “autonomous human choice” from the Individualism vs Collectivism Index (Hofstede et al., 2010), since it covers most of the countries we examined. Since the current evidence points towards the stability of national differences in individualism and autonomous choices over time (Schimmack et al., 2005), we treated it as a time‐invariant variable.

In its simplest formulation, postcommunism refers to the situation in which a country has ceased to be communist (Gill, 2003), sometimes adopting a capitalistic or neo‐capitalistic economy. Amongst OECD countries, those labeled as “postcommunist,” belong geographically to East Europe and some are classed as post‐Soviet states, since those states emerged from the collapse of the Union of the Soviet Socialist Republics. Our study includes the following postcommunist countries: Bulgaria, Croatia, Czech Republic, Estonia, Hungary, Latvia, Lithuania, Poland, Romania, and Slovakia.

### 2.4 Sampling, data, and analytical tools

The sampling technique used in this study followed a previously tested approach (Di Martino & Prilleltensky, 2020). Our main analyses relied on secondary data, which were drawn from several international sources (see Table 1).

**Table 1:** Descriptive statistics and information on life satisfaction, OECD SJI, and control variables

Variable	Statistics^a^	Description	Measurement	Source
Life satisfaction	6.49 (0.79)	Answer to the question: “*Please imagine a ladder, with steps numbered from 0 at the bottom to 10 at the top. The top of the ladder represents the best possible life for you and the bottom of the ladder represents the worst possible life for you. On which step of the ladder would you say you personally feel you stand at this time*?”	Continuous (from 0 to 10)	World Happiness Report
OECD SJI	6 (0.87)	Weighted OECD SJI subdomains: poverty prevention, equitable education, labor market access, social cohesion and nondiscrimination, intergenerational justice, and health	Continuous (from 0 to 10)	Bertelsmann Stiftung Institute
GDP per capita, PPP (current international $)	36.17 (17.31)	GDP per capita, current international dollars converted by PPP conversion factor	Continuous (expressed in billions of dollars)	World Development Indicators
Social capital	58.21 (11.95)	Legatum Prosperity Index pillar. Subdomains: personal & family relationships, social networks, interpersonal trust, institutional trust, civic & social participation	Continuous (from 0 to 100)	Legatum Prosperity Index
Postcommunism	‐	Countries that have transitioned from former communist political and economic governance	Binomial (Yes, No)	World Population Review
Yes	80 (24.39%)			
No	248 (75.6%)			
Autonomous human choice	57.51 (19.81)	Level of individualism versus collectivism measured according to Hofstede's cultural dimensions	Continuous (from 0 to 100)	Hofstede et al. (2010)

Abbreviations: GDP, gross domestic product; OECD, Organisation for Economic Co‐operation and Development; PPP, purchasing power parity; SJI, Social Justice Index.

^a^ Values expressed as frequency and percentage for categorical variables, and as mean with standard deviation for continuous variables.

The main sample is composed of aggregated cross‐sectional time‐series data, which were derived from 41 OECD countries, measured across the years 2009, 2011 and from 2014 until 2019, for a total of 328 macro‐level observations (see Table 2).

**Table 2:** Descriptive statistics for main variables across OECD countries and years

Country	Life satisfaction	OECD SJI	GDP per capita PPP (current international $)	Social capital	Autonomous human choice	Postcommunism
Australia	7.27 (0.07)	5.77 (0.08)	47,067.57 (4429.47)	67.31 (1.92)	90	No
Austria	7.22 (0.19)	6.29 (0.11)	49,781.66 (5199.76)	70.55 (2)	55	No
Belgium	6.93 (0.08)	6.26 (0.07)	45,837.16 (4832.64)	58.28 (1.69)	75	No
Bulgaria	4.76 (0.45)	4.63 (0.2)	17,295.75 (4145.79)	43.6 (2.87)	30	Yes
Canada	7.34 (0.11)	6.41 (0.12)	45,222.32 (3617.21)	71.62 (1.32)	80	No
Chile	6.5 (0.18)	4.83 (0.13)	21,070.48 (3499.07)	52.39 (1.99)	23	No
Croatia	5.4 (0.10)	5.17 (0.07)	22,558.8 (4166.24)	42.94 (1.52)	33	Yes
Cyprus	6.11 (0.49)	5.94 (0.16)	33,307.24 (3499.25)	52.31 (2.21)	NA	No
Czechia	6.7 (0.24)	6.71 (0.08)	32,547.65 (5798.40)	47.00 (2.54)	58	Yes
Denmark	7.62 (0.09)	7.46 (0.17)	50,722.86 (6576.52)	78.80 (1.51)	74	No
Estonia	5.69 (0.32)	6.23 (0.21)	28,607.67 (5337.6)	54.60 (2.86)	60	Yes
Finland	7.61 (0.22)	7.32 (0.05)	44,253.76 (4130.99)	73.93 (1.58)	63	No
France	6.56 (0.21)	6.42 (0.12)	41,133.83 (3800.11)	55.47 (1.85)	71	No
Germany	6.93 (0.2)	6.47 (0.18)	47,335.64 (5695.64)	67.11 (1.59)	67	No
Greece	5.40 (0.37)	4.89 (0.2)	27,189.38 (3330.42)	45.67 (2.26)	35	No
Hungary	5.41 (0.47)	5.93 (0.21)	25,266.51 (4681.92)	48.53 (2.44)	55	Yes
Iceland	7.5 (0.01)	7.84 (0.13)	51,385.61 (8878.47)	72.18 (1.75)	NA	No
Ireland	7.01 (0.08)	6.28 (0.2)	64,864.18 (16575.93)	68.92 (0.94)	70	No
Israel	7.23 (0.18)	5.26 (0.19)	35,946.23 (5109.16)	52.93 (2.73)	54	No
Italy	6.17 (0.23)	5.5 (0.1)	37,723.27 (3457.5)	53.62 (0.83)	76	No
Japan	5.93 (0.14)	5.51 (0.07)	38,959.23 (2922.97)	47.81 (1.98)	46	No
Latvia	5.63 (0.51)	5.32 (0.19)	23,651.44 (4967.78)	47.08 (1.86)	70	Yes
Lithuania	5.92 (0.36)	5.55 (0.27)	27,226.05 (6541.99)	38.97 (4.91)	60	Yes
Luxembourg	7.02 (0.18)	6.46 (0.15)	10,4106.14 (11894.87)	60.48 (3.91)	60	No
Malta	6.56 (0.24)	5.88 (0.16)	34,909.50 (6289.33)	65.98 (1.95)	59	No
Mexico	6.63 (0.26)	4.54 (0.2)	17,129.27 (3398.99)	45.49 (2.4)	30	No
Netherlands	7.45 (0.09)	7.06 (0.11)	51,037.28 (4300.37)	73.71 (1.97)	80	No
New Zealand	7.31 (0.08)	6.64 (0.09)	37,728.96 (4105.54)	75.81 (1.83)	79	No
Norway	7.53 (0.08)	7.64 (0.08)	63,907.78 (6858.68)	77.91 (1.8)	69	No
Poland	5.96 (0.23)	6.08 (0.22)	24,957.97 (5684.73)	50.71 (2.55)	60	Yes
Portugal	5.49 (0.36)	5.5 (0.3)	29,207.33 (3748.89)	53.49 (1.84)	27	No
Romania	5.78 (0.40)	4.66 (0.19)	21,246.11 (5564.55)	43.72 (3.16)	30	Yes
Slovakia	6.16 (0.15)	5.78 (0.2)	27,598.76 (4393.35)	47.71 (3.16)	52	Yes
Slovenia	6 (0.25)	6.55 (0.26)	31,805.13 (4389.21)	57.73 (2.02)	27	No
South Korea	5.97 (0.41)	5.15 (0.08)	34,494.96 (4008.19)	43.57 (2.09)	18	No
Spain	6.38 (0.12)	5.3 (0.13)	34,966.52 (3814.69)	58.04 (1.77)	51	No
Sweden	7.32 (0.06)	7.07 (0.12)	48,610.96 (4540.82)	72.98 (1.42)	71	No
Switzerland	7.51 (0.04)	6.37 (0.15)	64,971.95 (9538.97)	72.46 (1.51)	68	No
Turkey	5.35 (0.19)	4.64 (0.22)	22,013.32 (6829.56)	42.34 (6.67)	37	No
United Kingdom	6.92 (0.24)	6.43 (0.25)	41,751.40 (4095.6)	65.11 (0.99)	89	No
United States	6.99 (0.14)	5.1 (0.09)	56,842.59 (6121.55)	67.82 (1.5)	91	No
Total	6.49 (0.80)	6 (0.87)	39,176.59 (17318.19)	58.21 (11.95)	58	No
*n*	309	307	328	328	312	

*Note*: Life satisfaction, OECD SJI, GDP, and social capital scores are expressed as average values across time, with standard deviation in brackets; whereas autonomous human choice scores are invariant across time.

Abbreviations: GDP, gross domestic product; OECD, Organisation for Economic Co‐operation and Development; PPP, purchasing power parity; SJI, Social Justice Index.

To best analyze the panel data, we tested whether a pooled OLS regression would be more suitable than a Fixed effect model. Results from Wald test indicate that a fixed effect model is preferable to a pooled OLS, *F* = 29.75, *df* = (28, 245), *p* < 0.001. Therefore, to control for the effect of time, we regressed our main outcome variable (life satisfaction) onto the variable “time,” in each model considered.

Since our data set presented more than 5% of missing data on some variables such as OECD SJI and autonomous human choice, and Little's test (1988) showed that they were not missing completely at random (MCAR), *χ* ^2^_(14)_ = 91.97, *p* < 0.001, a decision was made to exclude them through listwise deletion. This resulted in the complete removal of every observation that presented a missing value at any of the time points considered (e.g., Bulgaria in 2009) and a consequent reduction of the sample size of about 15% (from 328 to 279 observations) in all models we tested.

Statistical analyses were conducted with the R v.4.0.3 software, with the support of the following packages: BaylorEdPsych v.05 (Beaujean & Beaujean, 2012), for Little's MCAR test (Little, 1988), Lavaan v.06‐7 for OLS multiple regression and Manifest Path Analysis (Rosseel, 2012); MVN v.5.8, for assessing univariate and multivariate normality (Korkmaz et al., 2014), car v3.0‐10, to draw histograms and Combined Conditional Expectations and RESiduals (CERES) plots (Fox et al., 2012), and nonnest2 for Vuong's test (Merkle et al., 2020). Microsoft 365 PowerPoint v. 2009 was used to draw the Manifest Path Analysis graph.

## 3 RESULTS

### 3.1 Data analysis and results

Results from the OLS multiple regression are in line with the ones obtained in an earlier study (Di Martino & Prilleltensky, 2020). Social capital proves once more to be the strongest predictor of life satisfaction, *β* = 0.49, *p* < 0.001, 95% BS CI (0.37, 0.62), followed by autonomous human choice, *β* = 0.16, *p* < 0.001, 95% BS CI (0.07, 0.24), OECD SJI, *β* = 0.11, *p* = 0.031, 95% BS CI (0.01, 0.22), postcommunism, *β* = −0.14, *p* = 0.001, 95% BS CI (−0.23, −0.06), and lastly GDP, *β* = 0.09, *p* < 0.001, 95% BS CI (0.04, 0.15). Time is the only nonsignificant variable associated to life satisfaction, *β* = 0.04, *p* = 0.135, 95% BS CI (−0.01, 0.10). In terms of *R* ^2^, the effects of OECD SJI and covariates explain around 73.3% of variance in LS.

As already mentioned, testing the second hypothesis required running a Manifest Path Analysis. Therefore, we built the recursive Model 2 on the structure of the previous model. In line with the principle of parsimony (Kline, 2016), we started first with the simplest model, by including only highest priority relationships between the main variables of interest. This entailed adding three additional parameters, which stemmed from regressing social capital, autonomous human choice, and GDP onto OECD SJI.

The hypothesized Model 2 failed to provide adequate fit, *χ* ^2^_(7)_ = 257.725, Bollen–Stine *p* < 0.001, CFI = 0.73, TLI = 0.422, RMSEA = 0.358, 90% CI (0.322, 0.396), SRMR = 0.137. An inspection of standardized residuals and modification indices revealed a series of unspecified regressions paths, particularly from postcommunism and autonomous human choice onto GDP and social capital. Therefore, a choice was made to respecify the model by adding those previously unspecified parameters, provided their established theoretical and statistical substantial contribution (Byrne, 2013). From a theoretical point of view, the literature seems to have found consensus around the positive impact of autonomous human choice on social capital and GDP (Allik & Realo, 2004; Welzel & Inglehart, 2010). Conversely, there is still an open debate between those who advocate a “weakness” in the social and economic structure of postcommunist countries (Howard, 2002) and those who contest those findings (Foa & Ekiert, 2017). Whilst addressing this debate would go beyond the scope of our study, it shows that the literature has investigated the theoretical and empirical links between postcommunism, social capital, and GDP.

**Figure 2:**
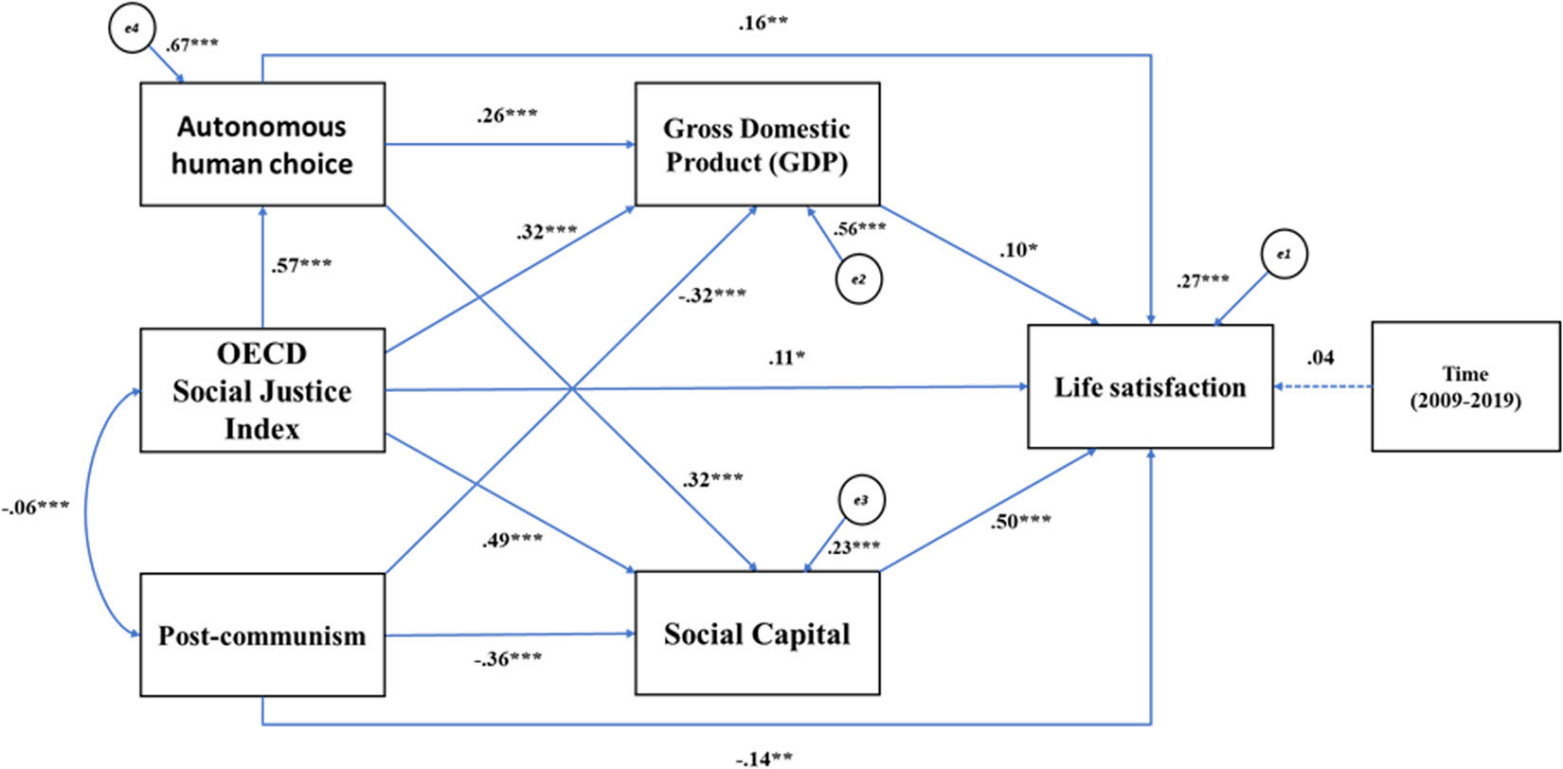
Model 3 Manifest Path Analysis for the effect of Organisation for Economic Co‐operation and Development (OECD) Social Justice Index on life satisfaction, multiple mediations between social capital, autonomous human choice, and gross domestic product (GDP), and control variables. *p < 0.05, **p < 0.01, ***p < 0.001. Manifest variables are in square boxes, measurement errors are in circles, one‐headed arrows represent direct effects, double‐headed arrows represent correlations between exogenous variables, dotted arrow indicates a nonsignificant effect based on *p* > 0.05

Statistical evidence offers additional support to the inclusion of those parameters, given their substantially large MI and EPC values. In fact, their inclusion substantially improves the fit of Model 3, *χ* ^2^_(5)_ = 13.265, *p* = 0.195, CFI = 0.992, TLI = 0.971, RMSEA = 0.077, 90% CI (0.027, 0.129), SRMR = 0.032, providing evidence that this is one of the possible models that we can apply to the data (Figure 2). In this model, only the value of the RMSEA is in the range of mediocre fit (MacCallum et al., 1996). However, as Kenny et al. (2015) have demonstrated, RMSEA tends to reject otherwise good fitting models in cases like Model 3 that have few degrees of freedom and relatively small sample size. Therefore, RMSEA cannot be considered completely reliable in this instance.

As we can see from Table 3 and Figure 2, the direct effect of our main variables on life satisfaction has not been substantially altered. In addition, the OECD SJI proves to be also a strong significant predictor of social capital, *β* = 0.49, *p* < 0.001, 95% BS CI (0.39, 0.56), autonomous human choice, *β* = 0.57, *p* < 0.001, 95% BS CI (0.49, 0.65), and GDP, *β* = 0.32, *p* < 0.001, 95% BS CI (0.26, 0.41). Moreover, social capital is also significantly predicted by autonomous human choice, *β* = 0.32, *p* < 0.001, 95% BS CI (0.21, 0.42), and postcommunism, *β* = −0.36, *p* < 0.001, 95% BS CI (−0.42, −0.30). Similarly, GDP is significantly predicted by autonomous human choice, *β* = 0.26, *p* < 0.001, 95% BS CI (0.13, 0.35), and postcommunism, *β* = −0.36, *p* < 0.001, 95% BS CI (−0.37, −0.28).

**Table 3:** Standardized coefficients, statistical significance, and confidence intervals of direct and indirect effects across tested models

	Model 1			Model 2			Model 3		
	*β*	*p*	CI	*β*	*p*	CI	*β*	*p*	CI
Direct effects									
*Predictors of life satisfaction*									
OECD SJI→LS (a)	0.11	0.02	0.01, 0.21	0.12	0.03	0.06, 0.25	0.11	0.02	0.006, 0.22
Social capital→LS	0.49	<0.001	0.37, 0.61	0.53	<0.001	0.43, 0.64	0.50	<0.001	0.37, 0.60
GDP→LS	0.09	<0.001	0.04, 0.15	0.10	<0.001	0.05, 0.15	0.09	0.01	0.03, 0.15
PC→LS	−0.14	0.001	−0.23, −0.05	−0.15	0.001	−0.22, −0.07	−0.14	<0.001	−0.23, −0.06
AUC→LS	0.16	<0.001	0.07, 0.24	0.17	<0.001	0.10, 0.24	0.16	<0.001	0.07, 0.24
Time→LS	0.04	0.14	−0.009, 0.10	0.05	0.13	−0.1, 0.11	0.04	0.13	−0.01, 0.10
*Social justice as predictor*									
OECD SJI→SC				0.74	<0.001	0.69, 0.78	0.49	<0.001	0.43, 0.55
OECD SJI→GDP				0.53	<0.001	0.48, 0.60	0.32	<0.001	0.26, 0.42
OECD SJI→AUC				0.57	<0.001	0.50, 0.64	0.57	<0.001	0.49, 0.65
*Autonomous human choice as predictor*									
AUC→SC				‐	‐	‐	0.32	<0.001	0.24, 0.38
AUC→GDP				‐	‐	‐	0.24	<0.001	0.13, 0.34
*Postcommunism as predictor*									
PC→SC				‐	‐	‐	−0.36	<0.001	−0.42, −0.31
PC→GDP				‐	‐	‐	−0.32	<0.001	−0.37, −0.28
*Total indirect effects*									
OECD SJI→GDP→LS (b)				‐	‐	‐	0.03	0.02	0.01, 0.05
OECD SJI→AUC→LS (c)				‐	‐	‐	0.09	<0.001	0.05, 0.14
OECD SJI→SC→LS (d)				‐	‐	‐	0.24	<0.001	0.17, 0.32
OECD SJI→AUC→ GDP→ (e)				‐	‐	‐	0.09	<0.001	0.06, 0.12
*Total direct and indirect effects combined*									
a + b + c + d + e				‐	‐	‐	0.58	<0.001	0.51, 0.65

Abbreviations: AUC, autonomous human choice; GDP, gross domestic product; LS, life satisfaction; OECD, Organisation for Economic Co‐operation and Development; PC, post‐communism; SC, social capital; SJI, Social Justice Index.

Lastly, it is worth noticing that the combined direct and indirect effects included in Model 3 explain around 72.7% of variance in life satisfaction, 76.1% in social capital, 43.5% in GDP, and 32.8% in autonomous human choice.

#### 3.1.1 Indirect and total effects

In terms of indirect effects, we found the strongest significant pathways going from OECD SJI to life satisfaction through social capital, *β* = 0.24, *p* = <0.001, BCa 95% BS CI (0.17, 0.32), followed by the path through autonomous human choice, *β* = 0.09, *p* = <0.001, BCa 95% BS CI (0.04, 0.14), and GDP, *β* = 0.03, *p* = 0.02, BCa 95% BS CI (0.009, 0.04).

We also found significant indirect pathways going from OECD SJI to life satisfaction, first through autonomous human choice and then through social capital, *β* = 0.09, *p* < 0.001, BCa 95% BS CI (0.06, 0.12), and through GDP, *β* = 0.01, *p* < 0.001, BCa 95% BS CI (0.005, 0.02).

The total of the combined direct and indirect effects of OECD SJI on life satisfaction shows a highly significant total effect, *β* = 0.48, *p* = <0.001, BCa 95% BS CI (0.39, 0.57).

**Figure 3:**
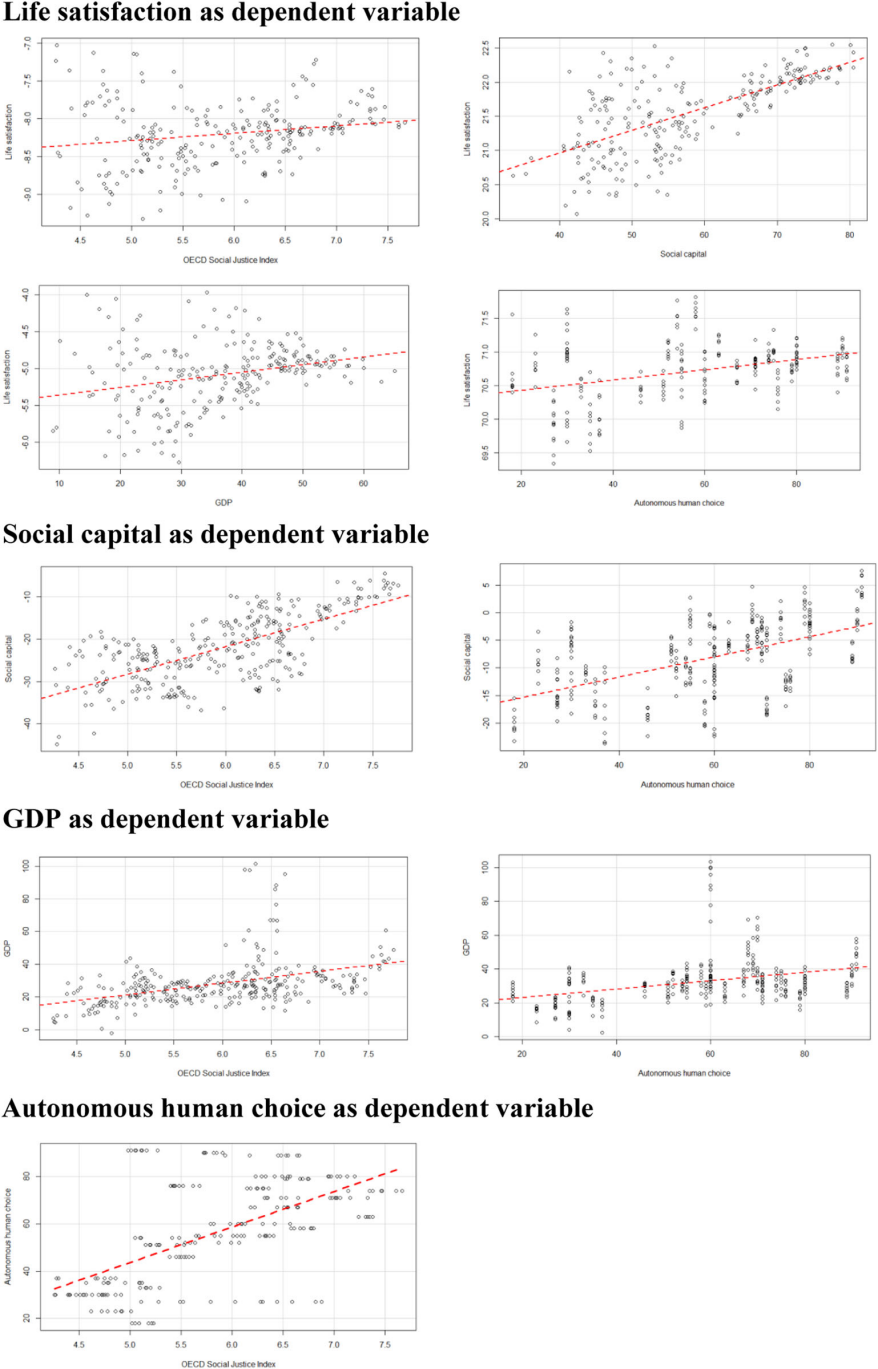
Combined Conditional Expectations and RESiduals plots. GDP, gross domestic product; OECD, Organisation for Economic Co‐operation and Development

The findings described above can also be inspected in a graphic format through a series of CERES plots (see Figure 3). These plots depict the effect of our predictors on their corresponding dependent variable, after controlling for the effect of the covariates (Cook, 1993).

Turning our attention to the unstandardized coefficients, we notice that each unit of increase in the OECD SJI (1 point on a scale from 0 to 10), is associated with a direct increase in national life satisfaction of about 0.1 points or 10% (on a scale from 0 to 10). On the other hand, each unit of increase in the OECD SJI is also associated with a direct increase in social capital of about 6.8 points (on a scale from 0 to 100), in autonomous human choice of about 13.67 points (on a scale from 0 to 100), and in GDP of about 7.14 billion dollars. In turn, life satisfaction shows an increase of about 0.3 points for every 10 points of increment in social capital, whereas 10 points of increment in autonomous human choice are associated with a direct increase in life satisfaction of about 0.06 points. Lastly, 1 billion increase in a country's GDP is associated with a direct increase of about 0.004 points in life satisfaction. Although we advise caution in drawing causal inferences from a correlational study, if our assumptions were correct, this would mean that that it might be necessary to raise the level of social justice in a country of about 4.5 points to make social capital increase life satisfaction of 1 point, all other values kept constant. On the other hand, raising a country level of social justice from its minimum to its maximum, or in other words of 10 points, would increase GDP of about 71.4 billion, which in turn would contribute only to 0.28 points of increase in life satisfaction. Similarly, an increase of 10 points in social justice would be likely to contribute of about 0.82 points of increase in life satisfaction through an increase in autonomous human choice.

#### 3.1.2 Cross‐validation and comparison of alternative models

Although rarely employed, cross‐validation and comparison of alternative models are always a desideratum in the analysis of covariance structures (Cudeck & Browne, 1983), and are particularly recommended when testing the validity of respecified models (Kline, 2016). To cross‐validate Model 3, we randomly partitioned our main sample into two sub‐samples, that is a calibration sample (168 observations, 127 used) and a validation sample (160 observations, 140 used). To avoid oversampling from a given time point, we adopted a stratified sample procedure, selecting 50% of cases from each year considered. Following Gana and Broc's (2019) recommendations, we then tested the measurement invariance of the two groups. The configural model showed acceptable model fit, *χ* ^2^_(8)_ = 10.992, Bollen–Stine *p* = 0.202, CFI = 0.997, TLI = 0.987, RMSEA = 0.052, 90% CI (0.000, 0.119), SRMR = 0.024. In addition, a nonsignificant *χ* ^2^ difference test shows that the calibration and validation sample can be considered invariant at the metric level, ΔMLR *χ* ^2^ = 6.47, Δ*df* = 14, Δ*p* = 0.06, and scalar level, ΔMLR *χ* ^2^ = 6.47, Δ*df* = 3, Δ*p* = 0.8, providing evidence that the two samples do not significantly vary with respect to intercepts and parameter estimates. These findings suggest that our main results could be replicated in other samples.

While being conscious that vast number of alternative and/or equivalent models could be compared against Model 3, we focused primarily on a selection of nonnested distinguishable models (see Vuong, 1989), in which OECD SJI is treated as endogenous variable, and the three main mediators used in Model 3, namely, social capital, GDP, and autonomous human choice, as exogenous variables (see Appendix B).

Looking at Table 4, we notice that most of the alternative models tested provide acceptable indices of model fit, and based on the Vuong's test results, most of them might also have equal fit in the population of interest. This indicates that they could be considered, at least from a statistical point of view, as possible alternatives to Model 3, although not necessarily a better one. Only in two cases, additional evidence suggests that two competing models could better fit data than Model 3. In fact, following Raftery's (1995) guidelines, a difference in the Bayesian Information Criterion (BIC) indices of 12.03 points between Model 3 and Model 5b tends to favor the latter model—which treats GDP as predictor of OECD SJI and autonomous human choice —as well as its equivalent version (Model 7)—which treats autonomous human choice as additional predictor of OECD SJI (see Appendix B).

**Table 4:** Comparisons of Model 3 with alternative and equivalent models

Model	*χ* ^2^_df_	CFI/TLI	RMSEA (CI)	SRMR	AIC/BIC	Vuong's test^a^
*OECD SJI as exogenous variable*						
Model 3	13.265_5_	0.99/0.97	0.07 (0.02, 0.12)	0.03	8867.613/8951.131	NA
*Social capital as exogenous variable*						
Model 4	85.301_6_	0.91/0.75	0.21 (0.17, 0.26)	0.06	8937.648/9017.535	H1A: *z* = 4.05, *p* < 0.001
						H1B: *z* = 4.05, *p* = 1
Model 4b	24.898_5_	0.97/0.92	0.15 (0.11, 0.19)	0.04	8896.117/8976.004	H1A: *z* = 1.31, *p* = 0.09
						H1B: *z* = 1.31, *p* = 0.90
*GDP as exogenous variable*						
Model 5	27.843_7_	0.97/0.94	0.10 (0.06, 0.14)	0.04	8878.191/8954.446	H1A: *z* = 1.36, *p* = 0.08
						H1B: *z* = 1.36, *p* = 0.91
Model 5b	6.866_6_	099/0.99	0.02 (0.00, 0.08)	0.02	8859.214/8939.101	H1A: *z* = −1.08, *p* = 0.86
						H1B: *z* = −1.08, *p* = 0.13
*Autonomous human choice as exogenous variable*						
Model 6	15.603_5_	0.99/0.96	0.08 (0.04, 0.13)	0.04	8869.951/8953.469	H1A: *z* = 0.43, *p* = 0.33
						H1B: *z* = 0.43, *p* = 0.66
Model 7	6.866_6_	0.99/0.99	0.02 (0.00, 0.08)	0.02	8859.214/8939.101	H1A: *z* = −1.08, *p* = 0.86
						H1B: *z* = −1.08, *p* = 0.1

Abbreviations: χ^2^, chi‐square test of model fit; AIC: Akaike Information Criterion; BIC: ‎Bayesian Information Criterion; CFI, comparative fit index; GDP, gross domestic product; OECD, Organisation for Economic Co‐operation and Development; RMSEA, root mean square error of approximation; SJI, Social Justice Index; SRMR, standardized root mean square residual; TLI, Tucker–Lewis index.

^a^ H1A: tests the alternative hypothesis that Model 3 fits better than the alternative model; H1B: tests the alternative hypothesis that the alternative model fits better than Model 3.

## 4 DISCUSSION

The findings presented here offer some additional evidence in support of the potential link between social justice and life satisfaction at the macro‐level of analysis. The results are encouraging if we consider that, although we sourced new data and used the OECD version of the SJI, our main findings are in line with the results obtained in a previous study (Di Martino & Prilleltensky, 2020).

In addition, in this article we were able to test some hypotheses that shed more light on a potential direct and indirect influence that social justice might exert on people's happiness at the national level. In Model 3, we tested whether the effect of social justice on national life satisfaction could be mediated by social capital, autonomous human choice, and GDP while controlling for covariates and other mediators. This hypothesis was strongly supported by good model fit indices and highly statistically significant parameters. While social capital shows the strongest direct effect on life satisfaction, it is in turn significantly predicted by the OECD SJI. Taking all the indirect and direct effects combined, the standardized regression coefficient of the effect of the OECD SJI on life satisfaction is around 0.58. If our model reflected the true relationship between those variables, such a value would deserve some attention, considering that past investigations have rarely included social justice amongst relevant predictors of life satisfaction at the national level.

Turning to the additional pathways we drew between our covariates, we provided statistically significant evidence in support of the hypothesis that autonomous human choice and postcommunism could be predictors of both social capital and GDP. In the final model we tested, while autonomous human choice proves to be positively linked to its target variables, postcommunism shows a negative link. These findings are in line with the literature (Allik & Realo, 2004), which supports the idea that postcommunist countries suffer from weaknesses in their social and economic structures (see Howard, 2002).

Although the mediating impact of autonomy on life satisfaction is much lower than that of social capital, it still deserves consideration. When taken in combination, we see that personal (autonomy), relational (social capital), and collective factors (social justice) are significant predictors of life satisfaction. This confirms long‐held beliefs in CP that these three sets of factors operate in synergy. Furthermore, they support the notion that social policies must attend to three sets of values concurrently: personal (freedom, autonomy, self‐determination, empowerment), relational (inclusion, compassion, trust, empathy, participation), and communal (justice, equality, fairness and access to resources) (Prilleltensky, 2001; Riemer et al., 2020; Watkins, 2019).

With regard to the epidemiological literature, this study expands the significant contributions made by Wilkinson and Pickett (2018). Whereas they looked primarily at economic inequality as a measure of injustice, our research incorporated a more capacious definition and construct of social justice, as measured by the multifactorial approach taken by the Bertelsmann Stiftung Institute.

Lastly, we believe that this study can contribute to the development of a psychosocial theory of the common good. For instance, it is possible to posit that conditions of justice lead to feelings of mattering, which, in turn, can make people feel valued and provide them an opportunity to add value. This sense of mattering, in turn, can promote wellness (Prilleltensky & Prilleltensky, 2021). We can further speculate that the more wellness people experience, the more they are able to contribute to the common good, sparking a virtuous cycle. In other words, this study can be considered a building block in the development of psychosocial foundations for well‐being beyond the personal level. Once that theory is tested, action can be generated to promote justice, autonomy, and relatedness, key elements of mattering.

## 5 LIMITATIONS AND FUTURE PERSPECTIVES

Some limitations of this study are worth reporting. The OECD SJI suffers from some statistical and theoretical shortcomings previously described for the EU SJI (Di Martino & Prilleltensky, 2020). From a theoretical point of view, although the index covers several aspects of social justice—including some innovative ones such as intergenerational justice—it lacks enough breadth on procedural justice. This is unfortunate since procedural justice has been strongly linked to the experience of happiness (Lucas et al., 2013).

In terms of measurement properties, we also need to point out that several components of the SJI ultimately are made to converge into a composite measure of social justice, which unfortunately lacks a direct statistical link with its underlying domains. This means that, amongst other things, there is no clear way to directly assess how strongly each component of the SJI is associated to life satisfaction. This has important implications for national policies, in that our findings are unable to provide clearer guidelines on which aspects of social justice governments need to improve to promote the happiness of their citizens.

This is also associated with the fact that the index uses a normative approach to measure social justice by putting together several indicators that are assumed to be equally accepted as aspects of social justice across 41 different OECD countries. Similar to other international indexes (e.g., Human Development Index, Fragile State Index, Legatum Prosperity Index), the OECD SJI presents a lack of contextualism and cross‐cultural sensitivity that should not be ignored when interpreting the results of this study.

Regarding our main findings, we should be mindful that these have been derived from a correlational study and as such we cannot exclude that some of the variables we modeled as endogenous could be in fact exogenous and vice versa. Although we provided some examples of alternative models, future studies should explore other candidate models, which could not be tested in this study. In particular, we recommend that future studies explore in more detail the alternative model in which GDP is treated as antecedent of the relationship between social justice and autonomous human choice and national happiness.

Additionally, we cannot exclude that unmodelled variables, other than social justice and postcommunism might contribute to explain variation in social capital, autonomous human choice, and GDP at the country level. In the same vein, there might be some unexplored predictors of life satisfaction, which were not included in this study.

In terms of sampling, one of the main limitations is that our data set includes only aggregated data at the country level. As such, we were unable to analyze our data through multilevel models as in the previous case (Di Martino & Prilleltensky, 2020). This prevented us from testing, among other things, the effect of individual‐level variables impacting on life satisfaction.

Additionally, having only 5 degrees of freedom and 279 observations, our main model (i.e., Model 3) reaches a power of 0.25, which is well below the recommended threshold of 0.8. Given the underpowered model, we cannot be confident in having escaped some Type II error. Lastly, the relatively small sample size combined with a limited number of time points prevented us from using longitudinal models to better analyze panel data.

As a last note of caution, we should bear in mind that our sample does not extend beyond OECD countries; therefore, we should be careful not to generalize these results to other countries.

## 6 CONCLUSIONS

In this paper, we provided quantitative evidence to support the direct link between social justice and life satisfaction at the national level. We have also investigated an indirect relationship between these two constructs, which is mediated by social capital, autonomy, and GDP. However, our findings warn us once more against considering GDP as the primary measure of country‐level wellness. Indeed, amongst all the variables employed in our study, GDP was the weakest link with national life satisfaction and the other variables we considered. While autonomous human choice had a positive relationship to social capital, GDP, and life satisfaction, postcommunism correlated negatively with all these variables. Our main findings show that promoting social justice is likely to increase aspects of social capital such as social relations, trust towards others and social institutions, and engagement in social life, all of which result in gains in life satisfaction. At the same time, equal opportunities and sound distributive policies might propel the economy of a country, with positive effects on citizens' satisfaction. Lastly, social justice has a positive impact on people's autonomous choice, which is another important element for the pursuit of a happy life.

These results suggest that the well‐being scholarship should consider higher‐level variables that are responsible for people's happiness. Beyond the individual, it is important to consider national‐level factors. Guided by CP's ecological perspective, we were able to offer evidence that confirms the interdependence among personal (autonomy), relational (social capital), and communal (social justice) determinants of wellness at the national level of analysis. Whereas prior epidemiological data elucidated the important role of economic inequality in well‐being and ill‐being, our study goes beyond financial disparities. The framework of social justice employed in the current study also includes a variety of factors impinging on distributive and procedural justice, which are directly and indirectly involved in promoting national life satisfaction.

Turning to the specific contribution that these findings make to the field of CP, our aim is to help the discipline expand its range of action towards the global arena (Marsella, 1998). Establishing a quantitative link between social justice, social capital, and autonomy is an attempt to inform CP of new ways to promote well‐being beyond the individual and community levels (Riemer et al., 2020). Although the field aspires to examine the full spectrum of ecological dynamics affecting well‐being, studies looking at national policies across time and place are missing. Although other disciplines have explored the role of freedom, agency, and emancipatory values on life satisfaction (Welzel & Inglehart, 2010), CP has not played a significant role in that scholarship. We believe that this study can help CP develop a psychosocial theory of the common good, which connects fairness with social, economic, cultural, and political factors that are related to national well‐being. We hope that our analyses will inspire action to promote personal, relational, and communal well‐being.

### PEER REVIEW

The peer review history for this article is available at https://publons.com/publon/10.1002/jcop.22822

## Data Availability

The data that support the findings of this study are available from the corresponding author upon reasonable request.
